# Supplementary material for: Binocular fusion enhances the efficiency of spot-the-difference gameplay
Source: PLoS One. 2021 Jul 20;16(7):e0254715. doi: 10.1371/journal.pone.0254715 (PMC8291752; doi:10.1371/journal.pone.0254715)
Supplement: Attachment — Submitted filename: reviewPlos.odt [file pone.0254715.s002.odt]

This study is definitely an interesting attempt at gamifying science but is not presented as such. However, the manuscript is more written as a game instruction manual than a scientific article. Results are very interesting but should be much better presented and put in perspective: Analysis is incomplete and results are not discussed. There is no discussion about which binocular mechanisms could be at play. What to do when authors claim their study would make a good Wikipedia entry? At times, this paper sounds very casual and non-scientific. It really looks as it was giving guidelines to play a game and not assessing the binocular mechanisms which enable better performance in fusion mode.


Presentation:

The formatting is really non-conventional and confusing. For instance, for table1: subjects details can be presented in a table as a function of the experiment. But aims and procedures should be clearly described in text. There are no “key features” in an experiment description. Each experiment should just be described in its own subsection. Generally speaking, I think it would have been better to present the paper experiment by experiment, explaining the stimuli and results together. 

Also, line 63: I didn’t check the journal policy, but I would suggest to avoid footnotes. This and the previous paragraph of the intro (line 52) makes the paper look not very academic. Actually, I think that a good description of rivalry and lustre effect is critical and should be fully integrated in the introduction.

Line 112: “Interestingly, the identification of local feature differences has been demonstrated to become more efficient when the companion images are rapidly presented one after the other in time, relative to when they are placed side-by-side.[13] The binocular fusion equivalent of this task – the present study – has, however, not been explored thus far.”
This is a very interesting point but it should be mentioned earlier, as part of the rationale, than after the list of the experiments. Comparing this serial presentation to your fusion protocol might also give interesting insights on the fusion mechanism to discuss in the discussion.

Even if we are not at the editing stage yet, I don’t think it is acceptable to submit figure as image file with so poor compression quality. Insets in figs 4 and 5 are not even readable.


Analysis:

There is no comprehensive analysis nor discussion of the data. The authors seem to have just performed ANOVAs without trying to understand the meaning of their data. This is particularly obvious for the analysis of exp4, figure6. What we can see from the figure:
there is no effect of polarity in the non fusion mode
fusion mode improves performance in the opposite polarity condition
fusion mode decreases performance in the same polarity condition.
However, there is no appropriate testing to demonstrate these points.

Also, in fig3b, the gameplay mode seems to be significant for the polarity and shape but not for the presence/absence condition. Appropriate post-hoc analysis should be performed to clarify these comparisons.


Discussion:

The discussion is not scientific by any means: Results are interesting and raises interesting questions which are not discussed at all in the discussion. 
In particular there is no discussion about why no effect of gameplay mode is observed for the presence/absence condition figure3. 
There is no discussion why no effect of contrast or colour saturation is observed (figs 4 and 5).
And there is no discussion about the polarity results either. Although these ones are particularly interesting as there is no difference in the non-fusion mode but a large difference in the fusion mode. I believe the same polarity condition is also the only condition for which performance in fusion mode is worse than in side-by-side mode?


Specific points:

Line 42: starting an article with “Figure 1A” is definitely non classical.

Line 133: “Images containing a given number of differences were presented three times, totaling up to 12 images for identification in each mode of gameplay”
Should it be images pairs?  3 times the same image? Please make sure to be accurate in the method description. The modes of gameplay should be introduced before.

Line 145: “Hence, a pilot experiment was conducted on 10 participants to divide the 24 images into two sets of 12 images each of equal complexity – one set for the fusion mode and the other set for the non- fusion mode (data not shown). These image sets were then used for the first experiment on a completely different set of participants.” The 2 sets should have been counterbalanced in a crossed design: each set should have been used in fusion mode for half of participants and non-fusion mode for the other half and oppositely. By the way I suggest the non fusion mode should be renamed “lateral” or “alongside” or side-by-side mode.

Fig2b, should be labelled “number of misses”

Figs 4 and 5: it would probably look better to illustrate the different contrasts by changing the luminance of the bars instead of using ugly patterns.

L336:” The main effects of color direction [F(2,38)=62.6, p=0.007, ηp 2 337 =0.24)] and chromatic saturation [F(3,57)=5.8, p=0.004, ηp 2=0.23)] and the interaction between these two factors  [F(6,114)=36.4, p2 338 =0.20)] were also statistically significant with large effect size”
The insets figure5 really seem to indicate the contrary. Please check your analysis and data. In case this is confirmed, you must present the data in a better way (likely showing individual data) to illustrate this claim.

Line 346: “Experiment 4: Is the advantage of fusion mode of gameplay lost with the “weakening” of binocular lustre? Yes, it does,” this sentence is very (too) casual.

Exp4: why naming the conditions “enhanced” and “weakened” lustre, you could just say same or opposite polarity. I had to go back to check in the methods what you meant by enhanced or weakened.

I don’t think the beginning of the first paragraph of the discussion has its place in a scientific article.

Line 385: “the present study could be cited as evidence in future edits of this Wikipedia entry!” Seriously?

2nd paragraph of the discussion: if your aim is just to play a game then I understand this paragraph and the previous paragraph. However, if your aim is to understand the science behind it, this paragraph has nothing to do there.
