# Supplementary material for: Binocular fusion enhances the efficiency of spot-the-difference gameplay
Source: PLoS One. 2021 Jul 20;16(7):e0254715. doi: 10.1371/journal.pone.0254715 (PMC8291752; doi:10.1371/journal.pone.0254715)
Supplement: Attachment — Submitted filename: Response to Reviewers.docx [file pone.0254715.s003.docx]

**Response to reviewers**

**Editorial Board Member**

**Both reviewers find the study and it's results interesting, but both note that the manuscript is at times lacking in scientific rigor. I particularly agree with both authors that the binocular mechanisms which underlie your findings need to be better discussed. Reviewer 1 further raises some methodological issues which should be addressed, and suggests that portions of the manuscript should be re-written to better fit a scientific publication. Finally, I cannot find anywhere the data underlying your results. Please note that Plos ONE policy requires your raw data to be publicly available, so you should either provide these data as supplementary information or upload them to a public data repository (e.g. Zenodo) and include the link to the deposit in your manuscript. *Response:*** *We thank the editor and the two reviewers for their insightful comments on the manuscript.* We have addressed all these comments systematically and they all appear as italicized text in the revised submission. Specifically, we have changed the following in the manuscript:

1. Re-organized the manuscript into four separate experiments with a dedicated methods, results and discussion section. All these experiments are tied together with a global introduction and discussion section.
2. All more details of the statistical tests in the results and interpret the results in the context of the underlying physiology of binocular vision and rivalry/lustre perception.
3. Removed all casual-sounding statements from the manuscript and replaced then (wherever applicable) with scientifically sound statements.
4. Re-organized the figures by removing the insets from Figures 2, 4 and 5 and include them as separate panels for ease of viewing.

We believe that these amendments have significantly improved the quality of our manuscript. We have also now made all the raw data of the study available to the readers by uploading an Excel sheet as supplementary information along with the revised version.

**Editorial office**

**Please ensure that your manuscript meets PLOS ONE's style requirements, including those for file naming.**

***Response:*** *We have gone through the guidelines for PLoS One style requirements and think we have met the requirements indicated there.*

**We note that you have included the phrase “data not shown” in your manuscript. Unfortunately, this does not meet our data sharing requirements. PLOS does not permit references to inaccessible data. We require that authors provide all relevant data within the paper, Supporting Information files, or in an acceptable, public repository. Please add a citation to support this phrase or upload the data that corresponds with these findings to a stable repository (such as Figshare or Dryad) and provide and URLs, DOIs, or accession numbers that may be used to access these data. Or, if the data are not a core part of the research being presented in your study, we ask that you remove the phrase that refers to these data.**

***Response:*** *That particular data does not form a core part of the research being presented in this study. We have therefore removed this phrase from the revised manuscript. In fact, based on the comments of the second reviewer, the entire segment containing this phrase has been modified to improve clarity of through process.*

**Thank you for stating the following financial disclosure: [The funders had no role in study design, data collection and analysis, decision to publish, or preparation of the manuscript.]. At this time, please address the following queries:**

a. **Please clarify the sources of funding (financial or material support) for your study. List the grants or organizations that supported your study, including funding received from your institution.**
b. **State what role the funders took in the study. If the funders had no role in your study, please state: “The funders had no role in study design, data collection and analysis, decision to publish, or preparation of the manuscript.”**
c. **If any authors received a salary from any of your funders, please state which authors and which funders.**
d. **If you did not receive any funding for this study, please state: “The authors received no specific funding for this work.”**

***Response:*** *The funding disclosures section of the manuscript now reads as follows “This study was supported by the Prof. Brien Holden Eye Research Centre, Hyderabad Eye Research Foundation. The funders of this study had no role in study design, data collection and analysis, decision to publish, or preparation of the manuscript. The funders of this study supported the salary of AH and SRB. The author(s) have no proprietary or commercial interest in any materials discussed in this article.”*

**Reviewer 1**

**This is a nice paper that makes a cute point about how spot-the-difference games can be quickly solved via binocular fusion of the difference-image pairs. Although the study does not provide any new theoretical insights into the mechanisms for detecting interocular differences in contrast, it has the merit of potentially appealing to a broad audience given the popularity of spot-the-difference gameplay. The paper is well-written, the methods sound and the results convincing. My only suggestion for improvement is that it would have been nice if the authors could say a little more about what are believed to be the underlying mechanisms for the detection of interocular contrast differences and what they think are the ecological situations in which these mechanisms are deployed by vision. There have been a number of recent papers on the subject of interocular contrast difference detection which a quick search will reveal and these could form the basis of a more general discussion of the topic.**

***Response:*** *We thank the reviewer for this comment. Based on the comments of the academic editor and the second reviewer, we have re-organized the manuscript into four different experiments with their own dedicated methods, results and discussion section. The underlying neurophysiology of binocular vision for the perception of luminance and chromatic lustre and how it may be applied to the spot-the-difference game are now included in the revised manuscript.*

**Reviewer 2**

**This study is definitely an interesting attempt at gamifying science but is not presented as such. However, the manuscript is more written as a game instruction manual than a scientific article. Results are very interesting but should be much better presented and put in perspective: Analysis is incomplete and results are not discussed. There is no discussion about which binocular mechanisms could be at play. What to do when authors claim their study would make a good Wikipedia entry? At times, this paper sounds very casual and non-scientific. It really looks as it was giving guidelines to play a game and not assessing the binocular mechanisms which enable better performance in fusion mode.**

***Response:*** *We thank the reviewer for their insightful comments on the manuscript.* *We have addressed all these comments systematically and they all appear as blue italicized text in the revised submission. Specifically, we have changed the following in the manuscript:*

1. *Re-organized the manuscript into four separate experiments with a dedicated methods, results and discussion section. All these experiments are tied together with a global introduction and discussion section.*
2. *All more details of the statistical tests in the results and interpret the results in the context of the underlying physiology of binocular vision and rivalry/lustre perception.*
3. *Removed all casual-sounding statements from the manuscript and replaced then (wherever applicable) with scientifically sound statements.*

*Re-organized the figures by removing the insets from Figures 2, 4 and 5 and include them as separate panels for ease of viewing.*

**Presentation:**

**The formatting is really non-conventional and confusing. For instance, for table1: subjects details can be presented in a table as a function of the experiment. But aims and procedures should be clearly described in text. There are no “key features” in an experiment description. Each experiment should just be described in its own subsection. Generally speaking, I think it would have been better to present the paper experiment by experiment, explaining the stimuli and results together.**

***Response:*** *We realize that the information presented in Table 1 (other than participant numbers, age-range and gender) are explained in detail in the methods section of the manuscript. To avoid redundancy, we have removed Table 1 from the revised manuscript. Information related to participant demographics are now included in the first paragraph of the Methods section of each experiment. The entire manuscript is also re-organized the manuscript into four separate experiments with a dedicated methods, results and discussion section. All these experiments are tied together with a global introduction and discussion section.*

**Also, line 63: I didn’t check the journal policy, but I would suggest to avoid footnotes. This and the previous paragraph of the intro (line 52) makes the paper look not very academic. Actually, I think that a good description of rivalry and lustre effect is critical and should be fully integrated in the introduction.**

***Response:*** *The footnote has been removed from the manuscript and the text related to binocular rivalry/lustre are now integrated into the second and third paragraphs of the revised introduction (lines 68 – 73). The first paragraph of the introduction is also modified to make the text more academically oriented (lines 48 – 58).*

**Line 112: “Interestingly, the identification of local feature differences has been demonstrated to become more efficient when the companion images are rapidly presented one after the other in time, relative to when they are placed side-by-side.[13] The binocular fusion equivalent of this task – the present study – has, however, not been explored thus far.” This is a very interesting point but it should be mentioned earlier, as part of the rationale, than after the list of the experiments. Comparing this serial presentation to your fusion protocol might also give interesting insights on the fusion mechanism to discuss in the discussion.**

***Response:*** *This sentence has now been moved to the second paragraph of the introduction section where a typical visual search task is described (lines 76 – 79). We agree with the reviewer that it would be interesting to compare the performance of subjects in the fusion task of the present study (where the two targets were presented simultaneously and fused using vergence eye movements) with that of the serial presentation paradigm by Josephs et al (2016). There is also the dichoptic equivalent of the Josephs et al paradigm where the companion images are serially presented one to each eye that may result in fusion depending on the time period of alternation. This could be achieved using efficiently using a shutter-goggle set-up. Presently, the lab does not have this set-up and it would take quite a bit of time to get this set up ready for experimentation. We would therefore not like to consider this experiment for the present study but reserve it for the future. A note about this comparison is, however, included in the discussion section of the revised manuscript (lines 345 – 356).*

**Even if we are not at the editing stage yet, I don’t think it is acceptable to submit figure as image file with so poor compression quality. Insets in figs 4 and 5 are not even readable.**

***Response:*** *The format for figures 2, 4 and 5 are now changed to show the results better. The insets have been removed and they are now represented in separate figure panels. The text has been modified accordingly.*

**Analysis:**

**There is no comprehensive analysis nor discussion of the data. The authors seem to have just performed ANOVAs without trying to understand the meaning of their data. This is particularly obvious for the analysis of exp4, figure6. What we can see from the figure:**

**-	there is no effect of polarity in the non fusion mode**

**-	fusion mode improves performance in the opposite polarity condition**

**-	fusion mode decreases performance in the same polarity condition.**

**However, there is no appropriate testing to demonstrate these points.**

**Also, in fig3b, the gameplay mode seems to be significant for the polarity and shape but not for the presence/absence condition. Appropriate post-hoc analysis should be performed to clarify these comparisons.**

***Response****: We have revised the description of the results for this section of the manuscript by including the following paragraph in the revised manuscript (lines 280 – 294). Qualitative observation of Figure 3B suggested that the fusion mode of gameplay decreased the time taken for identification for the polarity and shape differences but not for the presence/absence differences, all relative to the non-fusion mode of play (Fig 3B). This pattern was not appropriately captured in the three-factor RM-ANOVA described above for this dataset. Hence, two-tailed, heteroscedastic Student’s T-tests were performed with appropriate Bonferroni correction of p-values for multiple comparisons for each pair of fusion versus non-fusion gameplay mode data across different task complexities and difference types. The time taken for first identification was statistically significantly lesser in the fusion mode of gameplay relative to the non-fusion mode, across all task complexities for the polarity difference (mean difference range: 1.60 to 2.77sec; p<0.0001) (Fig 3B). The mean difference was smaller but statistically significant for shape differences for images containing 6, 9 and 12 differences (mean difference range: 1.23 to 2.5sec; p<0.0001) but not for 3 differences (mean difference: 0.27sec; p=0.06) (Fig 3B). For first identification of presence/absence differences, the difference between the fusion and non-fusion modes of gameplay were different only for images with 6 and 9 differences (mean difference range: 0.42 to 0.97sec; p≥0.02) but not for 3 and 12 differences (mean difference range: 0.11 to 0.14sec; p≥0.07) (Fig 3B).*

*We also note that appropriate post-hoc analyses have also now been reported for the luminance contrast and target size dataset in Experiment 2 (lines 391 – 397), for the color direction and color saturation dataset in Experiment 3 (lines 482 – 494) and for the same and opposite polarity dataset in Experiment 4 (lines 593 – 599). Taken together, we believe that the statistical reporting of our data is completer and more comprehensive than in the original submission.*

**Discussion:**

**The discussion is not scientific by any means: Results are interesting and raises interesting questions which are not discussed at all in the discussion. In particular there is no discussion about why no effect of gameplay mode is observed for the presence/absence condition figure 3. There is no discussion why no effect of contrast or colour saturation is observed (figs 4 and 5). And there is no discussion about the polarity results either. Although these ones are particularly interesting as there is no difference in the non-fusion mode but a large difference in the fusion mode. I believe the same polarity condition is also the only condition for which performance in fusion mode is worse than in side-by-side mode?**

***Response:*** *The manuscript is re-organized into four separate experiments and the discussion section of each experiment now contains a detailed analysis of the results obtained and the underlying neurophysiology that may have resulted in these results.*

**Specific points:**

**Line 42: starting an article with “Figure 1A” is definitely non classical.**

***Response:*** *The starting statement of the manuscript is rephrased as follows: “In a typical spot-the-difference game (Fig 1), differences in local features of two otherwise identical scenes are detected using an eye scanning (saccadic eye movements) and visual matching strategy.” (lines 48 – 49).*

**Line 133: “Images containing a given number of differences were presented three times, totaling up to 12 images for identification in each mode of gameplay”. Should it be images pairs? 3 times the same image? Please make sure to be accurate in the method description. The modes of gameplay should be introduced before.**

***Response:*** *Three different image pairs containing each of the aforementioned number of differences were created, totaling up to 12 image pairs for identification in the fusion and non-fusion modes of gameplay. The word “images” has been replaced with “image pairs” throughout the manuscript. The two modes of gameplay are also clearly indicated in the revised statement (lines 142 – 144).*

**Line 145: “Hence, a pilot experiment was conducted on 10 participants to divide the 24 images into two sets of 12 images each of equal complexity – one set for the fusion mode and the other set for the non- fusion mode (data not shown). These image sets were then used for the first experiment on a completely different set of participants.” The 2 sets should have been counterbalanced in a crossed design: each set should have been used in fusion mode for half of participants and non-fusion mode for the other half and oppositely. By the way I suggest the non fusion mode should be renamed “lateral” or “alongside” or side-by-side mode.**

***Response:*** *We realize that the description of the control experiment was not clear in the original submission. We have clarified our thoughts in the revised manuscript and this will hopefully address the reviewer’s comment (lines 155 – 162). Regarding the change in terminology for the game play modes, we see the reason for the reviewer’s suggestion as is more veridically describes the position of the companion images in the non-fusion mode of game play. However, since the terminologies of “fusion” and “non-fusion” directly describe the viewing experience of the study participants, we choose to retain this terminology in the revised manuscript.*

**Fig2b, should be labelled “number of misses”**

***Response:*** *This has been modified in the revised manuscript.*

**Figs 4 and 5: it would probably look better to illustrate the different contrasts by changing the luminance of the bars instead of using ugly patterns.**

***Response:*** *The patterns have been removed from both these figures. The different bar-pairs have now been identified with different saturations of the green color.*

**L336:” The main effects of color direction [F(2,38)=62.6, p=0.007, ηp 2 337 =0.24)] and chromatic saturation [F(3,57)=5.8, p=0.004, ηp 2=0.23)] and the interaction between these two factors [F(6,114)=36.4, p2 338 =0.20)] were also statistically significant with large effect size”. The insets figure5 really seem to indicate the contrary. Please check your analysis and data. In case this is confirmed, you must present the data in a better way (likely showing individual data) to illustrate this claim.**

***Response:*** *We have crossed checked the statistical analysis and we confirm the numbers reported for the main effects in the original manuscript. Two-tailed, heteroscedastic Student’s T-tests with appropriate Bonferroni correction of p-values for multiple comparisons indicated that the time taken for task completion was statistically significantly lesser in the fusion mode of gameplay relative to the non-fusion mode, across all color directions and chromatic saturations (p<0.001) (Fig 5B – D). Post-hoc pairwise comparisons for color direction revealed that the time taken for task completion in the two red-green color directions (140° – 320° and 170° – 350°) were not significantly different from each other (mean difference±1SEM =0.07±0.30sec; p>0.5) but both were significantly different from the blue-yellow color direction (64° – 244°; mean difference±1SEM =1.01±0.37sec; p=0.03), across all chromatic saturations and modes of gameplay. Post-hoc comparisons for chromatic saturation revealed that the time taken for task completion was significantly different only between the 12.5% and 25% color saturations (mean difference±1SEM =1.67±0.45sec; p=0.008), across all color directions and modes of gameplay. None of the other pairwise comparisons were significantly different from each other (p>0.2). These statements are now included in lines 482 – 494 of the revised manuscript.*

**Line 346: “Experiment 4: Is the advantage of fusion mode of gameplay lost with the “weakening” of binocular lustre? Yes, it does,” this sentence is very (too) casual.**

***Response:*** *This statement is modified in the revised manuscript.*

**Exp4: why naming the conditions “enhanced” and “weakened” lustre, you could just say same or opposite polarity. I had to go back to check in the methods what you meant by enhanced or weakened.**

***Response:*** *The usage of “enhanced” and “weakened” terminology is now removed from the revised manuscript. The text now describes the stimuli in Experiment 4 on the basis of their inter-pair polarity difference – opposite polarity or same polarity.*

**I don’t think the beginning of the first paragraph of the discussion has its place in a scientific article.**

***Response:*** *We agree with the reviewer that the first paragraph of the discussion section was written somewhat casually. We have now deleted all the statements in the Wikipedia entry and condensed the paragraph to simply indicate that claims about binocular fusion helping gameplay are available but scientific evidence is absent. We provide the necessary scientific evidence for this claim.*

**Line 385: “the present study could be cited as evidence in future edits of this Wikipedia entry!” Seriously?**

***Response:*** *This statement is now removed from the revised manuscript.*

**2nd paragraph of the discussion: if your aim is just to play a game then I understand this paragraph and the previous paragraph. However, if your aim is to understand the science behind it, this paragraph has nothing to do there.**

***Response:*** *We understand that this paragraph does not provide any insights into the science behind why fusion aids the identification of local feature differences. Based on the comments on the editor and the two reviewers, we have now added a separate paragraph describing a possible reason for this effect. The points made in the offending paragraph are however very relevant to the fusion mode of gameplay – in fact, they indicate instances where the fusion mode will not work to enhance gameplay efficiency. Hence, considering the broad audience base of PLoS One, we choose to retain this paragraph in the manuscript. We have, however, moved this paragraph as the penultimate one in the discussion section, thus reducing its importance in the sequence of things discussed.*
