# Supplementary material for: Binocular fusion enhances the efficiency of spot-the-difference gameplay
Source: PLoS One. 2021 Jul 20;16(7):e0254715. doi: 10.1371/journal.pone.0254715 (PMC8291752; doi:10.1371/journal.pone.0254715)
Supplement: Attachment — Submitted filename: Response to Reviewers.docx [file pone.0254715.s004.docx]

**Response to reviewers**

**Editorial Board Member**

**Reviewer 2 has confirmed that you have successfully addressed all their previous comments. Given the substantial restructuring of your paper, I think your final Discussion and Conclusion section is now appropriate for the journal. The reviewer has also provided some final suggestions regarding the interpretation of your study that I would ask you to consider and incorporate in your manuscript. I don't think these minor points should be controversial to address, so I will most likely assess your final revisions directly, with no need for further review. I look forward to receiving your revised manuscript.**

***Response:*** *We thank you and the second reviewer for the positive feedback on our manuscript. We have incorporated the changes suggested by the reviewer as blue italicized text in the manuscript. Based on your feedback, we are not modifying the general discussion of the revised manuscript.*

**Editorial office**

**Please review your reference list to ensure that it is complete and correct. If you have cited papers that have been retracted, please include the rationale for doing so in the manuscript text, or remove these references and replace them with relevant current references. Any changes to the reference list should be mentioned in the rebuttal letter that accompanies your revised manuscript. If you need to cite a retracted article, indicate the article’s retracted status in the References list and also include a citation and full reference for the retraction notice.**

***Response:*** *We have double-checked the reference list and we do not find any citations that need to be removed or amended.*

**Reviewer 2**

**I appreciate the efforts made by the authors and the new rearrangement of the manuscript. I still think the general conclusion reads more like a guide on how to optimally play a game than a scientific article. But I will let the editors judge if those are in line with the editorial policies of the journal. Otherwise, I only have minor issues regarding some points which I think should be discussed a bit more thoroughly in the sub-discussions parts.**

***Response:*** *We thank the reviewer for their positive comments on the manuscript. Based on the suggestions of the editor, we have not made any modifications to the general discussion section of the manuscript. The remaining suggestions are addressed and they appear as blue italicized text in the revised manuscript.*

**I think some hypothesis why no effect of contrast is observed for both luminance and color conditions should be discussed.**

***Response:*** *We wish to point out that such hypotheses were already included in the first revision of the manuscript. Lines 445 – 458 and 544 – 550 of the revised manuscript talk about these hypotheses for the contrast condition and the color condition, respectively.*

**Why the detection in the color condition is faster than the luminance condition should be discussed as well.
*Response:*** *We wish to point out the reasons has been discussed in lines 542 – 546 in the first revision of the manuscript.*

**Specific points:**

**Line 52: I don’t think people would play a game if they think it is laborious.**

***Response:*** *We have removed the word “laborious” from this statement in the revised manuscript (line 52).*

**Line 431: “The strength of binocular lustre is thought to be mediated by the amount of neuronal conflict between the ON and OFF-centered ganglion cells in the retina, when stimulated with luminance patches of opposite polarities.” And Line 636 “This blending-in may be the result of a weak or no neuronal conflict between the ON- and OFF-centered retinal ganglion cells when the inter-pair polarity of the local feature difference was in the same direction, relative to when they were of opposite polarity.” This is more likely to originate in V1 than in the retina.**

***Response:*** *The origin of the neuronal conflict being the ON- and OFF-centered ganglion cells was based on the paper by Wendt and Faul (2020). However, we agree that such conflicts may also originate at the visual cortical level. For the present study, the site of origin is inconsequential, as long as the conflict occurs. We have therefore modified the statements in the revised manuscript to indicate that the conflict may originate in either the retina or in the visual cortex (lines 435 – 438 and 647 – 650 in the revised manuscript).*

**Line 547: “The chances of experiencing chromatic lustre and, thus, the ease of identifying such image-pairs in the spot-the-difference game has been shown to increase with stimulus saturation and is expected to be somewhat lesser in the red-green color direction than in the blue-yellow direction.” Why?**

***Response:*** *We speculate that the increase in saturation in chromatic domain is akin to increase in contrast in luminance domain. Therefore, higher saturation among the dichoptic pairs might allow better increased strength of rivalry, this making the identifying of such pairs easier, compared to those with lower chromatic saturation. This is now made explicit in the revised manuscript (lines 554 – 559). The last component of the sentence “**and is expected to be somewhat lesser in the red-green color direction than in the blue-yellow direction****”*** *has been now removed, as it was a misinterpretation by us in the earlier version of the manuscript.*

**Line 671: you can’t say it is independent on those parameters. It is only independent in the range you tested.**

***Response:*** *We agree with the reviewer. We have added the phrase “…in the range tested here” to make the point clear (line 683 – 686 in the revised manuscript).*
